# Supplementary material for: Low use of condom and high STI incidence among men who have sex with men in PrEP programs
Source: PLoS One. 2021 Feb 4;16(2):e0245925. doi: 10.1371/journal.pone.0245925 (PMC7861516; doi:10.1371/journal.pone.0245925)
Supplement: S1 File — (PDF) [file pone.0245925.s002.pdf]

Información sobre el archivo: de la base resumida

Valores de las variables

Valor		Etiqueta
Edad_cat	1,00	20-25
	2,00	26-30
	3,00	31-35
	4,00	36-40
	5,00	>40
Edad_3cat	1,00	20-30
	2,00	31-40
	3,00	>40
ESPANOL_01	,00	Espanol
	1,00	No espanol
Origen_3cat	1,00	Esp
	2,00	Lat
	3,00	Otros
dism_uso_preserv_01	,00	No
	1,00	Si
PSTVOSEXOANALANTESPREP_3CAT	,00	Nunca
	1,00	<50%
	2,00	>50% + siempre
PSTVO_SEXO_ANAL_ ANTES_PREP_CAT	,00	Siempre + >50%
	1,00	Nunca + <50%
PSTVOSEXOANALDESPPREP_3CAT	,00	Nunca
	1,00	<50%
	2,00	>50% + siempre
Nº CONTACTOSSEXMESANTESPREP_3cat	,00	1-10
	1,00	10-50
	2,00	>50
NUM_CONTACTO_ SEX_MES_ANTES_ [illegible]	,00	<10
	1,00	>10
Nº CONTACTOSSEXMESDESDEPREP_3cat	,00	1-10
	1,00	10-50
	2,00	>50
DROGA_SINO	,00	No
	1,00	Si
DROGAS_SEX_SI_NO	,00	No
	1,00	Si
AUMNETO_CONSU_AL_ DROGAS_NUM	1	NO
	2	SI
ALCOHOL_NUM	2	NO
	3	SI
CANNABIS_NUM	2	NO
	3	SI
POPPER_NUM	2	NO
	3	SI
COCAINA_NUM	2	NO
	3	SI
EXTASIS_NUM	2	NO
	3	SI
MDMA_NUM	2	NO
	3	SI
GHB_NUM	2	NO
	3	SI

Valores de las variables

Valor		Etiqueta
MEFE_NUM	2	NO
	3	SI
TINA_NUM	2	NO
	3	SI
NOPREPEFECTP_ALC_DROGAS	2	NO
	3	SI
ALCOHOL_SEX_NUM	1	NO
	2	SI
CANNB_SEX_NUM	1	NO
	2	SI
POPPER_SEX_NUM	1	NO
	2	SI
COCAINA_SEX_NUM	1	NO
	2	SI
EXTASI_SEX_NUM	1	NO
	2	SI
MDMA_SEX_NUM	1	NO
	2	SI
GHB_SEX_NUM	1	NO
	2	SI
MEFE_SEX_NUM	1	NO
	2	SI
TINA_SEX_NUM	1	NO
	2	SI
APPS_NUM	1	NO
	2	SI
CHEMSEX_NUM	1	NO
	2	SI
NUM_EPIS_INI_AS_CAT	,00	0
	1,00	1
	2,00	2
	3,00	>2
NUM_ITS_6M_AS_CAT	,00	0
	1,00	1
	2,00	2
	3,00	>2
NUM_ITS_12M_AS_CAT	,00	0
	1,00	1
	2,00	2
	3,00	>2
NUM_ITS_18M_AS_CAT	,00	0
	1,00	1
	2,00	2
	3,00	>2
NUM_ITS_24M_AS_CAT	,00	0
	1,00	1
	2,00	2
	3,00	>2
filter_$	0	No seleccionado
	1	Seleccionado
