# Supplementary material for: Low use of condom and high STI incidence among men who have sex with men in PrEP programs
Source: PLoS One. 2021 Feb 4;16(2):e0245925. doi: 10.1371/journal.pone.0245925 (PMC7861516; doi:10.1371/journal.pone.0245925)
Supplement: Attachment — Submitted filename: Response to Reviewers.docx [file pone.0245925.s004.docx]

PONE-D-20-27294R1
Low use of condom and high STI incidence among men who have sex with men in PrEP programs

**Dear Editor,
We are very grateful for the feedback we have received.** **All the comments have been taken into consideration and we revised the manuscript accordingly. Responses to such comments point-by-point are sent below. We attached revised manuscript in two versions: one clean revised version and a version with track changes.
We remain at your disposal for any additional clarification.**

**Kind regards**

Minor comments:
1. Did any participants discontinue PrEP over the course of the follow-up? Or was the sample selected to include only those who stayed on PrEP for the duration of the study period? Either way, please include this information in the Methods section. **All participants selected stayed on PrEP during study period. Updated in methods section.**

2. Table 3 mentions "any distant metastases" under "CT" but this was not included in the Methods section. **Mistake updated, we would mean “Any location”.**

3. This manuscript would benefit for one more round of copy-editing for English grammar/style, especially in the Methods and Results sections. Some suggested revisions:
Methods:
- The sentence about eligibility criteria should read: "The eligibility criteria was taking PrEP, so all participants had sexual risk indications for this preventative measure as proposed by the guidelines [reference? More clarity on which guidelines needed here] and started taking PrEP at the first visit of the study." **Updated.**
- "sexualized drug use" instead of just "sexualized drugs" **Updated.**
Results:
- "the 85.4% (n=94) of participants" should just read "85.4% (n=94) of participants". There are a few other instances of this sentence structure in the same paragraph, which should be revised similarly. **Updated.**
- "...consumed by 94.5% of PrEP users" (missing the word of) **Updated.**
- some inconsistency with how % are reported, i.e. sometimes with (n=) and sometimes without. Please revise such that all are consistent and according to journal standards. **Updated.**
- "It is also determined the number of diagnosed STIs per individual." - I don't understand this sentence and it is not clear what result this is reporting.
**We wanted to make references to the number of STIs diagnosed, but this information is clear enough in Table 3, so this sentence has been removed from the results.**
